# Euthanasia in Mental Disorders: Clinical and Ethical Issues in the Cases of Two Women Suffering from Depression

**DOI:** 10.3390/healthcare13162019

**Published:** 2025-08-16

**Authors:** Giuseppe Bersani, Angela Iannitelli, Pascual Pimpinella, Francesco Sessa, Monica Salerno, Mario Chisari, Raffaella Rinaldi

**Affiliations:** 1 Faculty of Medicine, Sapienza University of Rome, Piazzale Aldo Moro 5, 00185 Roma, Italy; giuseppe.bersani@uniroma1.it; 2 Department of Biotechnological and Applied Clinical Sciences, University of L’Aquila, Via Vetoio, 67100 L’Aquila, Italy; iannitelliangela@gmail.com; 3 Jefe Cuerpo Médico Forense, Facultad de Ciencias Médicas, Universidad Nacional del Litoral, Santa Fé 3000, Argentina; ppimpinella@fcm.unl.edu.ar; 4 Department of Medical, Surgical and Advanced Technologies “G.F. Ingrassia”, University of Catania, 95121 Catania, Italy; monica.salerno@unict.it; 5 Faculty of Medicine and Surgery, “Kore” University of Enna, 94100 Enna, Italy; mario.chisari@unikore.it; 6 Department di Anatomical, Histological and Medico-Legal Sciences and of Locomotor System, “Sapienza” University of Rome, 00185 Roma, Italy; raffa.rinaldi@uniroma1.it

**Keywords:** euthanasia, assisted suicide, depression, psychiatric disorders, bioethics, informed consent, treatment resistance, personality disorders, psychoanalysis, medico-legal evaluation, end-of-life ethics

## Abstract

**Background/Objectives:** The extension of euthanasia and physician-assisted suicide to individuals with mental disorders presents a profound ethical, clinical, and legal challenge. While increasingly accepted in some jurisdictions, their application in psychiatric contexts—particularly in cases of depression—raises concerns about diagnostic precision, therapeutic adequacy, and the validity of informed consent. This study examines two controversial Belgian cases to explore the complexities of euthanasia for psychological suffering. **Methods:** A qualitative case analysis was conducted through a qualitative analysis of publicly available media sources. The cases were examined through clinical, psychoanalytic, and medico-legal lenses to assess diagnostic clarity, treatment history, and ethical considerations. No access to official medical records was available. **Case Presentation:** The first case involved a young woman whose depressive symptoms were reportedly linked to trauma from a terrorist attack. The second concerned a middle-aged woman convicted of infanticide and later diagnosed with Major Depression. **Discussion:** In both cases, euthanasia was granted on the grounds of “irreversible psychological suffering.” However, the absence of detailed clinical documentation, potential unresolved trauma, and lack of psychodynamic assessment raised doubts about the robustness of the evaluations and the validity of informed consent. **Conclusions:** These findings highlight the need for a more rigorous, multidisciplinary, and ethically grounded approach to psychiatric euthanasia. This study underscores the importance of precise diagnostic criteria, comprehensive treatment histories, and deeper exploration of unconscious and existential motivations. Safeguarding clinical integrity and ethical standards is essential in end-of-life decisions involving mental illness.

## 1. Introduction

The relationship between end-of-life medical practices—in particular, euthanasia and assisted suicide—and mental disorders is a highly sensitive, controversial, and complex issue, addressed and regulated in diverse ways across different cultural and legal systems [1]. The topic raises numerous unresolved ethical, clinical, and legal questions, particularly when the request for assisted death originates from individuals with psychiatric conditions.

The general bioethical implications of accepting a death request from a patient are already substantial, but they become even more critical when the request is made by a person suffering from a mental disorder. In such cases, the disorder itself may impair the individual’s ability to make autonomous decisions, thereby complicating access to end-of-life medical practices [2,3,4].

This paper critically evaluates the ethical and clinical justification for euthanasia in two cases of depressive disorders. Rather than providing an exhaustive case report, this article uses the two examples as a springboard for a broader exploration of the clinical, ethical, and psychoanalytic dimensions of euthanasia in the context of mental illness. The primary aim is to highlight the complexities and critical considerations that must inform such decisions, especially when psychiatric conditions are involved.

## 2. Methods

This study employed a qualitative, narrative, and thematic analysis of two publicly documented cases of euthanasia granted for depressive disorders. The analysis was conducted using clinical, psychoanalytic, and medico-legal lenses to explore diagnostic clarity, treatment history, and ethical considerations.

Given its lack of access to official medical records, this study relied exclusively on secondary media sources, which constitutes a significant methodological limitation. As no access to official clinical records or structured psychiatric interviews was available, diagnostic impressions were inferred from publicly reported symptoms and clinical labels. Therefore, standardized diagnostic frameworks such as DSM-5 or ICD-11 were not systematically applied, and any reference to specific disorders should be interpreted as provisional and illustrative. Nevertheless, the selected cases of Shanti De Corte and Geneviève Lhermitte were chosen due to their exceptional public visibility and the ethical controversy they generated. These cases are not statistically representative but are paradigmatic in illustrating the clinical and ethical tensions surrounding psychiatric euthanasia.

The narrative approach allowed for a contextualized understanding of each case, while thematic analysis facilitated the identification of recurring issues such as diagnostic ambiguity, treatment adequacy, and consent validity.

## 3. Case Presentation

### 3.1. Case 1

**Figure 1:**
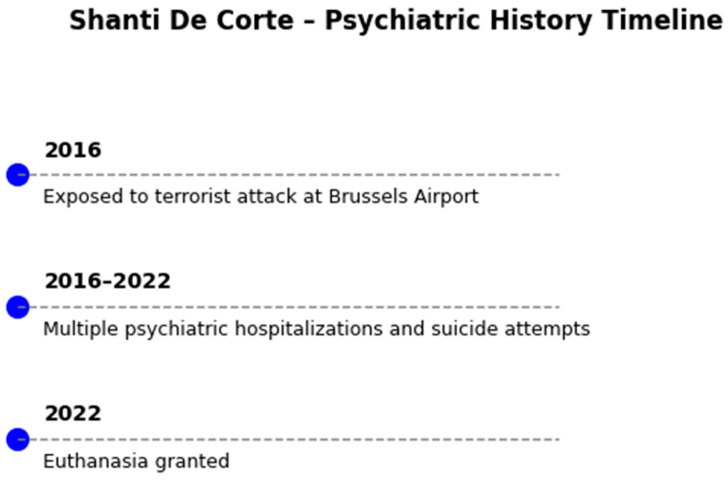
Timeline of key psychiatric events in the case of Shanti De Corte. This Figure illustrates the chronological sequence of major events based on publicly available sources: exposure to the 2016 Brussels Airport terrorist attack, subsequent psychiatric hospitalizations and suicide attempts between 2016 and 2022, and the approval of euthanasia in 2022.

In the first case, euthanasia was granted to a 23-year-old Belgian woman, Shanti De Corte, who had reportedly suffered for years from a depressive disorder following her exposure to a traumatic event—the 2016 terrorist attack at Brussels Airport, during which several of her friends were killed [5]. Her psychological suffering, described in media reports as persistent and unbearable, was ultimately deemed “incurable,” and thus sufficient grounds for approving her request for euthanasia under Belgian law (Figure 1).

However, the clinical details available are notably sparse and derived exclusively from press sources, as no access to official medical records was granted. The diagnosis of her condition remains unspecified in public accounts, though it has been hypothesized that she may have suffered from a combination of Post-Traumatic Stress Disorder (PTSD), Major Depressive Disorder, and possibly a comorbid Personality Disorder. Despite references to multiple psychiatric hospitalizations, suicide attempts, and pharmacological treatments, the nature, adequacy, and duration of these interventions remain unclear.

Of particular concern is the absence of a clearly documented psychiatric diagnosis and the lack of transparency regarding the therapeutic strategies employed. The patient reportedly described herself as taking “11 antidepressants,” a claim that, though likely exaggerated or misunderstood, raises questions about the appropriateness and coherence of her treatment plan. There is no mention in the media sources of evidence-based interventions such as electroconvulsive therapy (ECT), repetitive transcranial magnetic stimulation (rTMS), or ketamine treatment, which are considered effective in treatment-resistant depression. Their absence in the reporting may suggest a treatment gap, although this cannot be confirmed without access to clinical records.

Moreover, the decision to grant euthanasia appears to have been based primarily on the patient’s subjective report of “constant and unbearable psychological suffering,” without sufficient corroboration from objective clinical assessments. This raises critical questions about the evaluation of her capacity to provide informed consent, especially given that suicidal ideation is a core symptom of severe depression and may reflect a distorted perception of reality rather than a rational, autonomous decision.

This case also highlights a broader ethical dilemma: whether a request for euthanasia in the context of a severe mental disorder should be interpreted as a symptom of the illness itself rather than as a valid expression of self-determination. The lack of psychiatric engagement in the public discourse surrounding this case further underscores the need for a more robust, multidisciplinary approach to such profoundly consequential decisions.

In summary, the case of Shanti De Corte exemplifies the complexities and potential pitfalls of extending euthanasia to individuals with psychiatric conditions. It underscores the necessity for rigorous diagnostic clarity, comprehensive treatment histories, and careful ethical scrutiny—particularly when the request for death may itself be a manifestation of the underlying disorder.

### 3.2. Case 2

**Figure 2:**
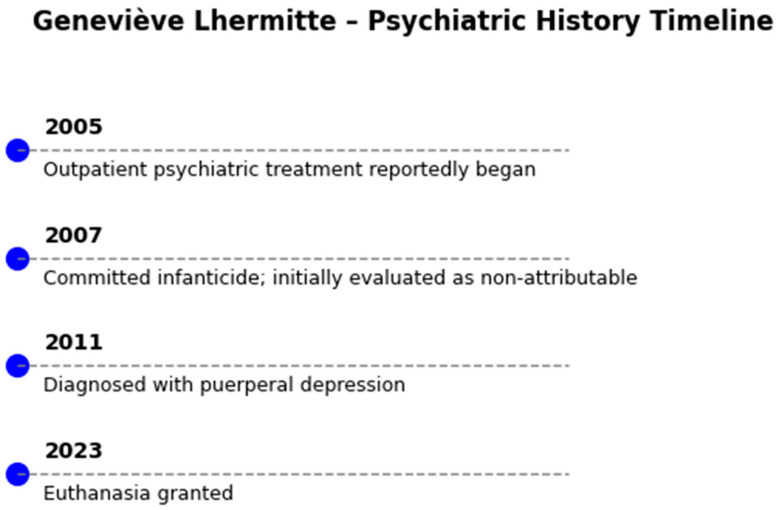
Timeline of key psychiatric events in the case of Geneviève Lhermitte. This Figure outlines major milestones based on publicly available sources, including the onset of outpatient psychiatric treatment in 2005, the infanticide incident in 2007, a diagnosis of puerperal depression in 2011, and the approval of euthanasia in 2023.

In the second case [6], euthanasia was granted to Geneviève Lhermitte, a 56-year-old Belgian woman who had been convicted of the infanticide of her five minor children. The murders, committed in 2007, were carried out in a premeditated and ritualistic manner, and she was sentenced to life imprisonment. Despite an initial psychiatric evaluation suggesting non-attributability due to mental illness, the court ultimately held her criminally responsible. In the years following her conviction, Lhermitte was transferred to a psychiatric facility under semi-liberty. In 2023, she was granted euthanasia on the grounds of “irreversible psychological suffering” (Figure 2).

This case is particularly complex, raising profound clinical, ethical, and legal questions. The available information, primarily from media sources, indicates that Lhermitte had a history of psychiatric vulnerability, including a diagnosis of puerperal depression in 2011 and outpatient psychiatric treatment dating back to 2005. However, there is no clear documentation of a sustained psychiatric diagnosis or treatment plan following the infanticide. The lack of transparency regarding her psychiatric care, both before and after the crime, leaves significant gaps in understanding the trajectory of her mental health.

The circumstances of the murders—including the methodical killing of each child, the symbolic placement of their bodies, and the presence of dissociative or hallucinatory experiences—suggest a severe psychiatric breakdown, possibly involving psychotic depression or dissociative states. Yet, the court’s decision to sentence her to life imprisonment, despite psychiatric recommendations, introduces a contradiction: she was deemed mentally ill enough to be considered non-attributable, but was still held legally accountable.

This contradiction becomes even more striking when juxtaposed with the later acceptance of her euthanasia request. If her mental illness was severe enough to justify euthanasia, was her capacity for valid and informed consent thoroughly evaluated? Conversely, if she had recovered sufficient insight to make such a request autonomously, does this not imply a remission of the psychiatric condition that had once rendered her non-attributable?

The ethical implications are equally troubling. It is plausible that her request for euthanasia stemmed from overwhelming guilt and remorse, rather than from a persistent, treatment-resistant depressive disorder. If so, the suffering may have been existential rather than pathological, raising questions about whether euthanasia is an appropriate response to moral anguish. Moreover, the media sources do not provide detailed information about the therapeutic interventions she received, such as pharmacological treatments, psychotherapy, or advanced interventions like ECT, rTMS, or ketamine. The lack of such references may indicate that these options were not pursued, but this remains speculative in the absence of clinical documentation.

From a medico-legal perspective, the case illustrates the ambiguity in Belgian legislation, which permits euthanasia for psychological suffering without requiring a clearly defined psychiatric diagnosis. This legal permissiveness, while intended to respect autonomy, may inadvertently allow for decisions that bypass rigorous clinical scrutiny.

In summary, the case of Geneviève Lhermitte exemplifies the ethical and clinical tensions inherent in granting euthanasia for mental disorders. It underscores the need for the following:

- Transparent diagnostic criteria;
- Thorough documentation of treatment history;
- Robust assessments of decision-making capacity; and
- A multidisciplinary approach that includes psychiatric, ethical, and legal expertise.

Without these safeguards, there is a risk that euthanasia may be granted in contexts where the suffering, though profound, might still be amenable to treatment—or where the request itself may be a symptom of the very disorder it seeks to escape.

## 4. Discussion

To discuss completely the two selected cases, Table 1 outlines the clinical, ethical, and legal dimensions. This table highlights key differences in psychiatric history, diagnostic transparency, treatment adequacy, and the complexities surrounding informed consent and ethical justification.

In both cases, the request for euthanasia was based on the existence of a “condition of irreversible psychological suffering”. A related clinical issue concerns the management of treatment-resistant depression (TRD), a condition often underlying requests for psychiatric euthanasia. TRD is defined by the failure of at least two adequate antidepressant trials and requires careful diagnostic and therapeutic evaluation. Esketamine, recently approved by regulatory agencies for TRD, has shown promising results, though it remains surrounded by misconceptions regarding its safety, efficacy, and dissociative effects. Clarifying these misunderstandings is essential to ensure that patients with severe depression receive all viable therapeutic options before considering end-of-life decisions [7]. Recent real-world evidence further supports the effectiveness of esketamine nasal spray (ESK-NS) in TRD, not only through clinician-rated outcomes but also from the patients’ perspective. In a three-month observational study, patients reported significant improvements in depressive symptoms, anhedonia, suicidality, and sleep quality, with some differences in timing compared to clinician assessments. These findings underscore the importance of integrating patient-reported outcomes into routine evaluation, as they may reveal nuanced therapeutic effects and inform more personalized treatment strategies [8]. In addition to clinical efficacy, recent machine learning applications have explored predictors of response to ESK-NS in TRD patients. A multicentric real-world study involving 149 individuals identified features such as severe anhedonia, anxious distress, mixed symptoms, and bipolarity as positive predictors of response and remission. Conversely, high depression severity and benzodiazepine use were associated with delayed improvement. These findings highlight the potential of predictive modeling in guiding personalized treatment strategies and optimizing esketamine use in clinical practice [9]. A recent Delphi survey conducted among Italian psychiatrists further confirmed the heterogeneity in TRD management across the country. While a consensus was reached on the importance of augmentation strategies and long-term maintenance, esketamine nasal spray emerged as the most promising option for TRD. Notably, clinicians agreed on its feasibility in outpatient settings, provided adequate patient education is ensured [10].

In retrospect, this raises significant ethical, clinical, and medico-legal concerns, particularly regarding the criteria used to assess irremediability and consent capacity.

The two cases differ substantially in clinical history, personal background, and environmental context. However, they share critical commonalities that allow for a unified evaluation of the methodological and ethical fragilities in the decision-making process. In both instances, the diagnoses—broadly labeled as “depressive disorders”—were not clearly defined or supported by comprehensive clinical documentation. Moreover, both cases involved trauma-related psychopathology: in the first, linked to a terrorist attack; in the second, to the aftermath of infanticide. Despite these complexities, there is no evidence that structured diagnostic frameworks, such as DSM-5 or ICD-11 criteria, were rigorously applied.

The notion of “irreversible suffering” and therapeutic futility was central to the approval of euthanasia in both cases. However, the criteria for establishing irremediability, particularly in psychiatric conditions, remain ambiguous and inconsistently applied [11]. While the decisions appear to align with prevailing practices in Belgium and other jurisdictions where psychiatric euthanasia is legal [12,13,14], they also expose gaps in clinical rigor and ethical safeguards.

### 4.1. Euthanasia in Mental Disorders: Clinical Considerations

A fundamental clinical question arises: is mental suffering being treated as a standalone justification for euthanasia, or is it being properly contextualized within a diagnosable psychiatric disorder? If the former, the scope of euthanasia could dangerously expand to include any form of existential distress. If the latter, then precise diagnostic criteria, treatment history, and prognosis must be thoroughly evaluated.

In both cases, the lack of detailed clinical records leaves open the possibility that the psychiatric evaluations were superficial or ideologically biased [15].

The distinction between chronic and treatment-resistant depression is crucial but appears to have been overlooked. No information is available regarding the use of advanced treatments such as ECT, rTMS, or ketamine, which are often effective in refractory cases.

Moreover, the subjective nature of suffering must be balanced with objective clinical assessments. Accepting a patient’s self-report without corroborating evidence risks medicalizing despair and undermining the therapeutic imperative [16].

There is a well-founded concern that ideological leanings may have influenced clinical judgments, rather than evidence-based psychiatric evaluation [17].

### 4.2. Psychoanalytic Considerations

Although the psychoanalytic literature on euthanasia remains limited, the growing prevalence of assisted death has prompted deeper inquiry into its unconscious motivations [18]. Suicide, a central theme in psychiatry, is often ambivalent and dynamic, influenced by internal conflicts that may not be consciously accessible.

Studies show that over 60% of individuals requesting assisted death have a diagnosis of depression or another mental disorder [19,20], and that such decisions are often unstable over time, influenced by symptom relief and therapeutic support [21]. This indicates that such requests might arise from transient emotional states, not enduring, autonomous choices.

The psychoanalytic framework emphasizes the internal conflict between life and death drives, as described by Freud and later theorists [22,23,24,25]. Freud’s concept of the death drive (Todestrieb) suggests an unconscious pull toward self-destruction, particularly when the ego is overwhelmed by unresolved trauma, guilt, or affective dysregulation [23]. Melanie Klein further elaborated on these dynamics, emphasizing the role of early object relations and persecutory anxieties in suicidal tendencies [26].

In this light, the euthanasia requests of Shanti De Corte and Geneviève Lhermitte may have been symptomatic expressions of unresolved trauma, guilt, or dissociation, rather than rational decisions. The presence of depressive or dissociative symptoms in both cases may reflect unconscious mechanisms such as introjected aggression, self-punishment, or the collapse of the containing function of the ego [27,28,29]. A psychodynamic evaluation might have uncovered unconscious factors that could have altered the clinical trajectory or at least introduced a therapeutic pause. For example, psychodynamic psychotherapy has been shown to reduce suicidal ideation by fostering insight into unconscious conflicts and enhancing emotional regulation [30,31,32].

Incorporating a psychoanalytic lens into the evaluation of euthanasia requests, especially in psychiatric cases, may serve as a protective factor against premature conclusions of irremediability. This perspective invites clinicians to explore the symbolic meaning of the death wish and to differentiate between autonomous will and expressions of psychic pain that are amenable to treatment [33,34].

### 4.3. Ethical Considerations

The most pressing ethical issue concerns the capacity to provide valid consent. In Belgium, the assessment of decision-making capacity in euthanasia cases is governed by the Belgian Act on Euthanasia of 2002. For psychiatric patients, the law requires that the request be voluntary, well considered, and repeated, and that the patient be legally competent. In addition to the attending physician, at least two other physicians must be consulted, one of whom must be a psychiatrist. These professionals are tasked with evaluating the patient’s mental state and capacity to make an informed decision. However, the law does not mandate the use of structured diagnostic tools or standardized instruments (e.g., MacCAT-T or MINI) for assessing capacity. This reliance on clinical judgment introduces variability and potential subjectivity, particularly in complex psychiatric cases. The absence of uniform protocols underscores the need for clearer guidelines and the integration of validated assessment tools to ensure ethical consistency and safeguard patient autonomy [35].

In severe depression, the desire for death is often a symptom, not a rational choice. Thus, a euthanasia request may signal the need for intensified care, not its cessation [36]. This creates a paradox: only those with milder depression may be deemed competent to request euthanasia, yet their suffering may not meet the threshold of irremediability. Conversely, those with severe depression may lack the capacity to consent yet are the most likely to request death. This tension challenges the ethical coherence of psychiatric euthanasia.

Furthermore, the role of personality disorders must be considered [37]. Chronic emotional suffering associated with borderline or other personality disorders may amplify the perception of incurability, both for the patient and the clinician [38,39]. In such cases, depressive symptoms may be more treatable than perceived, but the underlying personality structure complicates the clinical picture. This dynamic may have been at play in the first case, where no evidence of mood oscillation, a hallmark of primary mood disorders, was reported [40]. The absence of such features raises questions about whether the depressive state was secondary to a deeper personality pathology, and whether this was adequately assessed. Emerging neurophenomenological insights suggest that esketamine-induced dissociation may have therapeutic value in depression subtypes marked by depersonalization and reduced interoceptive awareness. According to the “relaxed prior hypothesis,” ketamine enhances sensitivity to unexpected bodily and emotional signals, promoting short-term psychological plasticity and supporting the view that dissociation, rather than being merely adverse, may facilitate therapeutic transformation by opening a transient window for affective recalibration [41]. Beyond symptomatology and treatment response, the role of affective temperaments (ATs) has gained increasing attention in understanding mood disorders. Originally conceptualized by Kraepelin and later refined by Akiskal, ATs are considered stable, subclinical traits that may predispose individuals to specific psychiatric conditions. Evidence suggests that ATs influence not only the onset and course of mood disorders but also treatment adherence, suicidality, and stress coping. Incorporating temperament assessment into psychiatric evaluation may enhance diagnostic precision and support more personalized therapeutic strategies [42].

These cases underscore the need for a unified framework that integrates the clinical, ethical, and psychoanalytic dimensions in evaluating euthanasia requests for psychiatric conditions. From a clinical standpoint, rigorous diagnostic procedures and comprehensive treatment histories are essential to assess therapeutic futility. Ethically, the principles of autonomy, beneficence, and non-maleficence must be carefully balanced. Autonomy requires that patients are capable of making informed, voluntary decisions, yet severe depression may compromise this capacity. Beneficence and non-maleficence demand that clinicians act in the patient’s best interest, avoiding harm while alleviating suffering. Psychoanalytically, unconscious motivations such as guilt, trauma, or dissociation may influence the desire for death, suggesting that such requests should be explored through a psychodynamic lens before irreversible decisions are made.

These insights have important implications for clinical practice and policy. Guidelines should mandate multidisciplinary evaluations, including psychiatric, ethical, and psychotherapeutic input, before approving euthanasia for mental disorders. Policies should also require documentation of treatment-resistant status, structured assessments of decision-making capacity, and consideration of alternative therapies. By embedding these safeguards, healthcare systems can better navigate the ethical complexities of psychiatric euthanasia while protecting vulnerable individuals.

Although this paper critically evaluates the ethical and clinical justification for euthanasia in two widely publicized cases of depressive disorders, it has several limitations. A key methodological limitation is the exclusive reliance on secondary media sources, as access to original clinical records was not available. Additionally, the absence of direct clinical interviews, legal documentation, and first-person accounts limits the depth of the analysis. Media narratives may also introduce bias or omit critical clinical and legal details. Furthermore, the retrospective and interpretative nature of the study, combined with the exceptional nature of the cases, restricts the generalizability of the findings to broader psychiatric populations or policy contexts.

## 5. Conclusions

The analysis of these two cases highlights the profound complexity underlying requests for euthanasia in the context of mental disorders. In both instances, depressive pathology played a central role in motivating the request for assisted death. However, the mere presence of a depressive disorder introduces additional layers of clinical, ethical, and legal complexity that must be carefully considered when evaluating the legitimacy and acceptability of such requests.

Two core elements emerge as critical in this evaluation: the objective recognition of suffering associated with the mental disorder, and the rigorous assessment of the individual’s capacity to formulate a valid and autonomous request. These elements must be addressed alongside the broader ethical concerns that inherently accompany any decision involving the intentional termination of life.

Importantly, these concerns are not limited to depression alone. They extend, albeit with necessary clinical distinctions, to other psychiatric conditions that may similarly give rise to requests for euthanasia. Despite their relevance, such cases remain under-represented in the broader discourse on end-of-life care, underscoring the need for greater attention and nuanced analysis.

To ensure ethical and clinical rigor in psychiatric euthanasia, we recommend the implementation of specific safeguards. These include the mandatory use of structured diagnostic tools (e.g., DSM-5 or ICD-11 criteria), comprehensive documentation of treatment resistance, and the requirement of at least one independent psychiatric second opinion. Multidisciplinary review boards should be involved in all cases, integrating psychiatric, ethical, and legal expertise.

In addition, future policy frameworks should consider the following safeguards:

- the mandatory use of structured diagnostic interviews and validated tools for assessing decision-making capacity;
- the requirement for longitudinal documentation of treatment resistance, including trials of evidence-based interventions such as ECT, rTMS, and ketamine;
- the inclusion of psychodynamic and existential assessments to explore unconscious motivations and symbolic meanings behind the euthanasia request;
- the establishment of national registries to monitor psychiatric euthanasia cases and ensure transparency; and
- the development of international consensus guidelines to harmonize ethical standards and reduce variability across jurisdictions.

These measures would help ensure that euthanasia decisions in psychiatric contexts are made with the highest degree of clinical rigor, ethical integrity, and procedural accountability.

Future research should focus on longitudinal studies assessing the stability of euthanasia requests in psychiatric populations, the role of unconscious motivations, and the effectiveness of emerging treatments such as esketamine. Additionally, there is a pressing need for the development of international guidelines that harmonize ethical standards and clinical procedures, ensuring consistency and protection for vulnerable individuals across jurisdictions.

Ultimately, any consideration of euthanasia in psychiatric contexts should be firmly based on a comprehensive, multidisciplinary assessment that integrates the personal, psychological, clinical, and ethical dimensions. Failure to recognize underlying psychopathological factors or to critically interrogate the influence of ideological or procedural biases risks leading to decisions with irreversible and ethically fraught consequences.

## Figures and Tables

**Table 1:** Comparative summary of two Belgian euthanasia cases involving psychiatric suffering.

Aspect	Case 1: Shanti De Corte	Case 2: Geneviève Lhermitte
Age	23	56
Background	Survivor of 2016 Brussels Airport terrorist attack; suffered psychological trauma	Convicted of infanticide (killed five children in 2007); sentenced to life imprisonment
Psychiatric History	Suspected PTSD, Major Depressive Disorder, possible Personality Disorder; multiple hospitalizations and suicide attempts	History of psychiatric vulnerability; puerperal depression (2011); outpatient treatment since 2005
Diagnosis Transparency	No official diagnosis disclosed; media-based speculation	No sustained or clearly documented diagnosis post-crime
Treatment History	Unclear; claimed to take “11 antidepressants”; no evidence of ECT or rTMS	Unclear; no detailed record of pharmacological or psychotherapeutic interventions
Grounds for Euthanasia	“Unbearable and incurable psychological suffering”	“Irreversible psychological suffering”
Legal Status at Time of Euthanasia	Civilian	In psychiatric facility under semi-liberty after life sentence
Consent Concerns	Questions about capacity due to depression and suicidal ideation	Questions about whether guilt/remorse influenced decision; contradiction with earlier legal accountability
Ethical Issues	Lack of diagnostic clarity; possible misinterpretation of suicidal ideation as autonomy	Tension between legal responsibility and psychiatric vulnerability; existential vs. pathological suffering
Transparency and Oversight	Sparse clinical details; no access to medical records	Limited information on psychiatric care and treatment trajectory
Key Concerns	Subjective suffering not corroborated by objective assessments; unclear treatment adequacy	Possible untreated or inadequately treated psychiatric condition; moral anguish vs. mental illness
Broader Implications	Need for rigorous diagnostic and ethical standards in psychiatric euthanasia	Highlights ambiguity in Belgian law and need for multidisciplinary review

## Data Availability

No new data were created or analyzed in this study. Data sharing is not applicable to this article.

## Abbreviations

The following abbreviations are used in this manuscript:

PTSD	Post-Traumatic Stress Disorder
ECT	Electroconvulsive Therapy
rTMS	Repetitive Transcranial Magnetic Stimulation
DSM-5	Diagnostic and Statistical Manual of Mental Disorders, 5th Edition
ICD-11	International Classification of Diseases, 11th Revision
